# Embolizing the middle meningeal artery to treat chronic subdural hematoma: a systematic review and meta-analysis

**DOI:** 10.3389/fsurg.2026.1941903

**Published:** 2026-08-21

**Authors:** Kuijia Duan, Rongmei Xia, Jibo Lv

**Affiliations:** 1 Department of Neurosurgery, Qujing Central Hospital of Yunnan Province (Qujing First People's Hospital), Qujing, Yunnan, China; 2 Department of Anesthesiology, Qujing Central Hospital of Yunnan Province (Qujing First People's Hospital), Qujing, Yunnan, China

**Keywords:** chronic subdural hematoma, embolization, meta-analysis, middle meningeal artery, systematic review

## Abstract

**Background:**

Comparative studies on the middle meningeal artery (MMA) embolization versus conventional management (CM) to treat chronic subdural hematoma (CSDH) yield inconsistent findings.

**Objective:**

This systematic review sought to meta-analyze studies comparing MMA embolization versus CM for treating CSDH.

**Methods:**

Terms including “hematoma”, “subdural”, “chronic”, “meningeal arteries”, “embolization”, and “therapeutic” were applied to search PubMed, Embase, the Cochrane Library, and Web of Science databases from their inception to July 11, 2025. After independent reading, screening, and evaluation by two researchers, data were analyzed and processed using RevMan v5.4.

**Results:**

Initially, 1,883 articles were retrieved. After eliminating 692 duplicates and excluding 1,087 articles that are conference abstracts, letters, or have ineligible research objectives, interventions, or outcomes, 104 articles were identified as potentially eligible. These articles were subsequently screened according to the eligibility criteria, and 20 studies were ultimately included. According to the meta-analysis, in the 20 studies involving 255,057 patients, 181,993 (71.4%) and 73,064 (28.6%) patients received MMA embolization and CM, respectively. MMA embolization performed better than CM in terms of treatment failure (relative risk [RR] = 0.40 [0.18–0.87], *P* = 0.007), recurrence rate (RR = 0.43 [0.32–0.57], *P* = 0.15), and complication rate (RR = 0.91 [0.77–1.08], *P* = 0.06). No difference was observed between the two groups for mortality (RR = 0.72 [0.55–0.94], *P* = 0.94), treatment failure rate (RR = 0.40 [0.18–0.87], *P* = 0.007), surgical salvage rate (RR = 0.59 [0.22–1.56], *P* = 0.29), rehospitalization (RR = 0.66 [0.41–1.06], *P* = 0.24), and improvement rate (RR = 1.05 [0.79–1.41], *P* = 0.43).

**Conclusion:**

MMA embolization may lower the failure rate of treatment and cut the demand for surgical salvage without elevating the risk. Therefore, MMA embolization may be considered in the management of CSDH.

**Systematic Review Registration:**

https://www.crd.york.ac.uk/PROSPERO/myprospero, PROSPERO CRD420251090976.

## Introduction

As the population ages progressively, the surgical management of chronic subdural hematoma (CSDH) is on the rise across the globe, particularly in China. CSDH is classified into traumatic and non-traumatic types based on whether there is a history of trauma. In addition, they can also be classified into non-separated, separated, unilateral, and bilateral types according to morphological features on imaging. In the context of a super-aged society, overcoming this disease continues to pose a formidable public health challenge. Although our understanding of the etiology of the disease and surgical techniques has evolved significantly, treatment outcomes remain suboptimal, with high recurrence rates persisting (1). Throughout medical history, renowned experts have grappled with identifying the optimal therapeutic regimen for this condition and have long debated its etiology. In 1657, CSDH was initially documented by Wepere, who described it as “delayed apoplexy.” In 1857, the term “pachymeningitis hemorrhagica interna” was first coined by Virchow, who believed that inflammation contributes significantly to the formation of CSDH. In 1914, the primary etiology of CSDH was identified by Trotter to be trauma.

Current evidence suggests that an inflammatory response to subacute or chronic SDH induces the formation of vascularized membranes, which impede resorption and contribute to recurrence (2, 3).

In 1883, a successful case of trepanation for CSDH was reported by Hulke. Subsequently, the use of various surgical approaches for hematoma removal, including burr holes and craniotomy, was documented, with novel techniques introduced worldwide in the ensuing decades. Putnam and Cushing reported a case series of 50 patients undergoing craniotomy in 1925. Horrax and Poppen's reports (1935–1937) on favorable outcomes following saline irrigation via burr holes marked a turning point, establishing this technique as the preferred alternative to craniotomy for CSDH. Later on, the twist drill technique was introduced. In 1988, Karakhan first reported the endoscopic evacuation of CSDH. Embolization of the middle meningeal artery (MMA) was first documented in 2000 by Mandai et al., who used polyvinyl alcohol particles to prevent recurrence. Initially, this technique served as a supplement to standard surgical intervention for recurrent CSDH, administered either preoperatively or postoperatively.

Overall, the preferred treatment for CSDH is still simple burr hole evacuation. However, there is growing interest in twist drill and the emerging hollow screw for CSDH among older patients owing to their reduced invasiveness (4). For hematomas that are recurrent or tricky, endoscopic intervention represents a preferred approach (5). MMA embolization has recently shown promise as an approach for managing both primary and recurrent CSDH. This endovascular procedure has been increasingly employed for CSDH treatment (6). It has exhibited good safety and efficacy in CSDH treatment, particularly among patients contraindicated for surgical intervention or those at substantial risk of recurrence (1, 7).

By 2030, it is estimated that CSDH will result in a minimum of 60,000 new cases per year within the United States (8). Symptoms of CSDH at presentation, such as cognitive decline, memory impairment, incontinence, diminished mobility, and particularly impaired consciousness, are more prevalent among older patients (9–11). CSDH represents a prevalent clinical condition manifesting as blood accumulation in the subdural space. While literature reports a broad range of CSDH incidence (1.7–20.6 per 100,000), this figure escalates substantially to 127.0 per 100,000 in the population aged over 80 years (12).

## Methods

### Study selection criteria

Comparative studies published in English were selected following the PICO (Patient, Intervention, Comparison, and Outcome) criteria:

1. Patients: individuals affected by CSDH;
2. Intervention: standalone or adjunctive MMA embolization;
3. Comparison: standard therapeutic regimens, comprising conservative or surgical management (e.g., twist drill craniostomy [TDC], burr hole drainage [BHD], and craniotomy);
4. Outcomes: treatment failure (hematoma thickness or residual hematoma after treatment ≥ 10 mm), recurrence (resolved or cured after treatment, but hematoma thickness exceeded 10 mm after burr-hole drainage with the passage of time), surgical salvage (need for urgent surgical reintervention, worsening of clinical condition), complication (medical complications such as infection, bleeding, and infarction), mortality, rehospitalization, and improvement rate (mRS reduction, hemmorhage thickness, and radiological resolution).

Non-English publications, review articles, non-comparative studies, case reports, and letters were excluded.

### Search strategy

We searched the Medline, PubMed, Embase, the Cochrane Library, and Web of Science databases from their inception to July 11th, 2025 for accessible studies utilizing reasonable combinations of Medical Subject Headings (MeSH) terms. These terms included: (“hematoma, subdural, chronic” [MeSH Terms] OR “chronic subdural hematoma” [Title/Abstract] or (“Subdural Hematoma, Chronic” [Title/Abstract] or “Hematoma, Chronic Subdurall” [Title/Abstract] or “Hematomas, Chronic Subdural” [Title/Abstract] OR “chronic subdural hematomas” [Title/Abstract] OR (“Subdural Hematomas, Chronic” [Title/Abstract]) and (“meningeal artery” [Title/Abstract] OR “Meningeal Arteries” [Title/Abstract] OR “arteria meningealis” [Title/Abstract] OR “MMA” [Title/Abstract]) and Embolization, Therapeutic [MeSH Terms]. Other keywords used for search included meningeal arteries, embolization, therapeutic, surgery, burr-hole, and craniotomy (Supplementary Table S1).

Medline, PubMed, Embase, the Cochrane Library, and Web of Science databases were searched up to July 11th, 2025, using subdural hematoma and embolization as the MeSH terms. The complete search strategy is provided in the Supplementary Materials.

### Data extraction

Two independent researchers (Kuijia Duan and Rongmei Xia) read the eligible articles, collected data into two separate Excel spreadsheets, and then cross-checked the spreadsheets. Each spreadsheet contained the following information: author name, publication year, country of origin, study design, sample size, age, sex, trauma history, antiplatelet or anticoagulant use, hematoma laterality, and hematoma width.

### Risk-of-bias assessment

Methodological quality of studies included in this systematic review was evaluated utilizing the Cochrane risk-of-bias tool for RCTs and the Newcastle-Ottawa Scale (NOS) for retrospective observational studies.

### Statistical analysis

All meta-analyses were performed using RevMan v5.4 (The Cochrane Collaboration). All statistical tests were two-tailed, with *P* < 0.05 indicating statistical significance. The relative risk (RR) of categorical data and mean difference (MD) of continuous data with the corresponding 95% confidence interval (CI) were pooled via the Mantel-Haenszel and inverse-variance tests, respectively. If statistical heterogeneity was assessed as not significant, data were pooled across studies via a fixed-effects model. If the I^2^ value exceeded 50%, indicating statistically significant heterogeneity, a random-effects model was applied. Funnel plots were constructed to assess publication bias.

### Quality of evidence

Certainty in evidence was evaluated via the Grading of Recommendations Assessment, Development, and Evaluation (GRADE) approach using GRADEpro GDT online (https://gdt.gradepro.org/app) (Supplementary Table S2).

## Results

### Study selection

**Figure 1:**
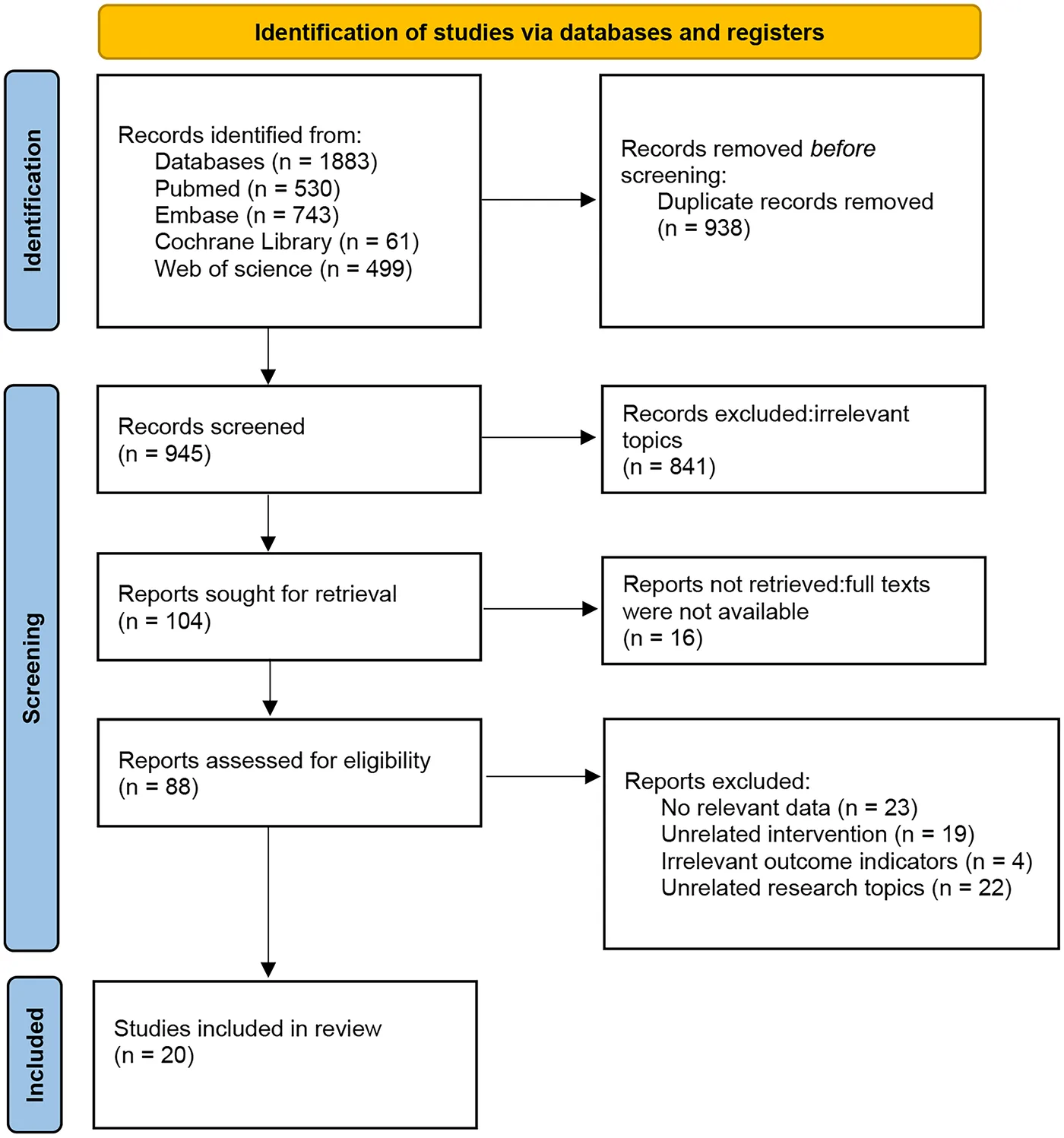
Literature screening flowchart.

The search yielded 1,883 records, comprising 530 from Medline and PubMed, 743 from Embase, 61 from the Cochrane Library, and 499 from Web of Science, of which 938 duplicate records were discarded. The remaining 945 articles underwent title and abstract screening, and 841 studies with irrelevant topics were excluded. Among the remaining 104 studies, full texts were not available for 16 studies, leaving 88 studies potentially eligible. Subsequently, studies were further excluded for no relevant data (*n* = 15), ineligible intervention (*n* = 19), ineligible outcome indicators (*n* = 4), and irrelevant research topics (*n* = 22). Ultimately, 20 studies were included in this review. The screening flowchart is depicted in Figure 1.

Published between 2017 and 2025, the included studies originated from the United States (*n* = 7), France (*n* = 3), South Korea (*n* = 2), Germany (*n* = 2), Australia (*n* = 1), Brazil (*n* = 1), Thailand (*n* = 1), and China (*n* = 3). These studies include 5 RCTs, 11 retrospective cohort studies (single-center), 3 prospective studies, and 1 pilot study. These studies enrolled 255,057 participants affected by CSDH, of whom 181,993 (71.35%) and 73,064 (28.6%) received conventional management (CM) and MMA embolization, respectively. The mean age ranged from 67 to 80 years across studies. Characteristics of the studies are listed in Supplementary Table S3.

### Risk-of-bias assessment

**Figure 2:**
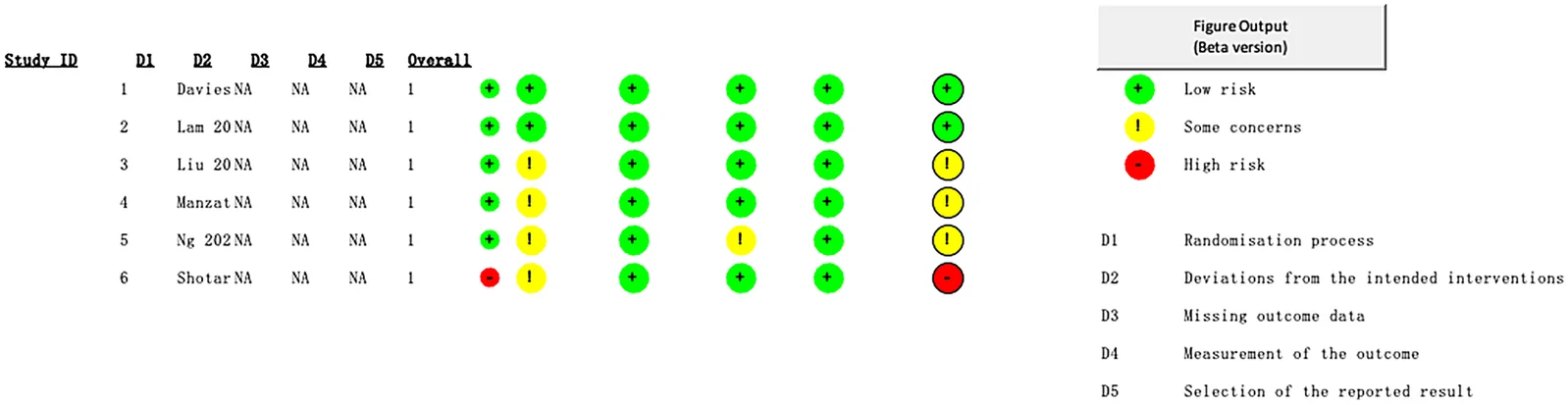
Cochrane risk-of-bias assessment.

The risk of bias was assessed utilizing the Cochrane risk-of-bias tool for RCTs (Figure 2) and NOS for non-randomized studies (Supplementary Table S4).

### PICO

Refer to Supplementary Table S5 for details of PICO and follow-up duration in each included study.

### Meta-analysis of the outcomes

#### Recurrence rate

**Figure 3:**
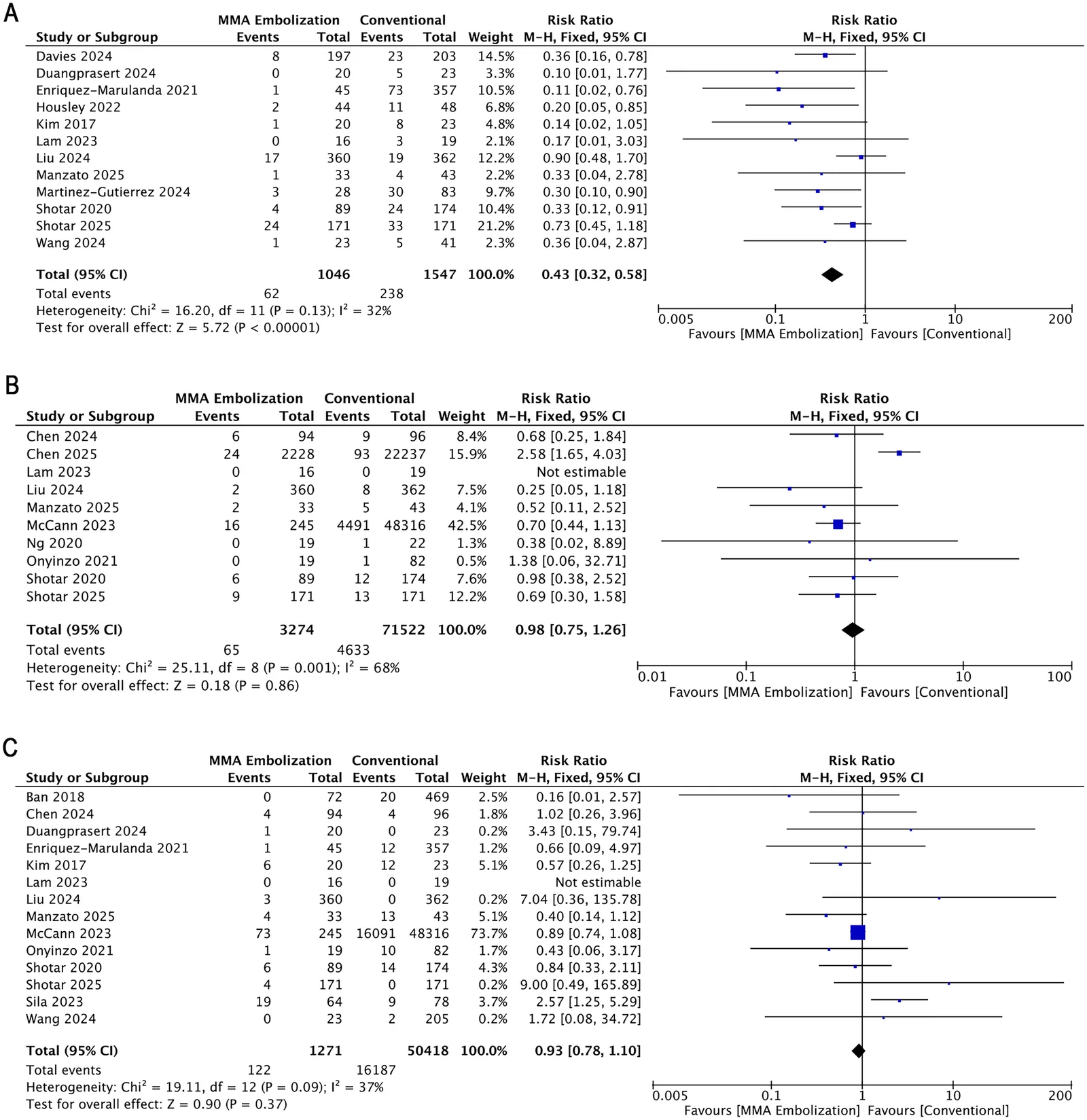
Forest plot for **(A)** recurrence rate; **(B)** complications; **(C)** mortality.

Twelve studies reported recurrence rate, with 62 events in the MMA embolization group (*n* = 1,046) and 238 events in the CM group (*n* = 1,547). The RR of recurrence rate was significantly lower following MMA embolization compared with CM (5.9% versus 15.4%, RR = 0.43 [0.32,0.58], I^2^ = 32%, *P* < 0.00001; Figure 3A and Supplementary Figure S1).

#### Complications

Fourteen studies reported complications, with 122 events in the MMA embolization group (*n* = 1,271) and 16,187 events in the CM group (*n* = 50,418). The RR of complications was significantly lower following MMA embolization compared with CM (9.6% versus 32.1%, RR = 0.93 [0.78,1.10], I^2^ = 37%, *P* = 0.37; Figure 3B and Supplementary Figure S2).

#### Mortality

Ten studies reported mortality, with 65 and 4,633 events in the MMA embolization (*n* = 3,274) and CM (*n* = 71,522) groups, respectively. No difference in mortality risk was observed between the groups (2.0% versus 6.4%, RR = 0.98 [0.75–1.26], *P* = 0.86, I^2^ = 68%; Figure 3C and Supplementary Figure S3). The certainty of the evidence was low.

#### Treatment failure

**Figure 4:**
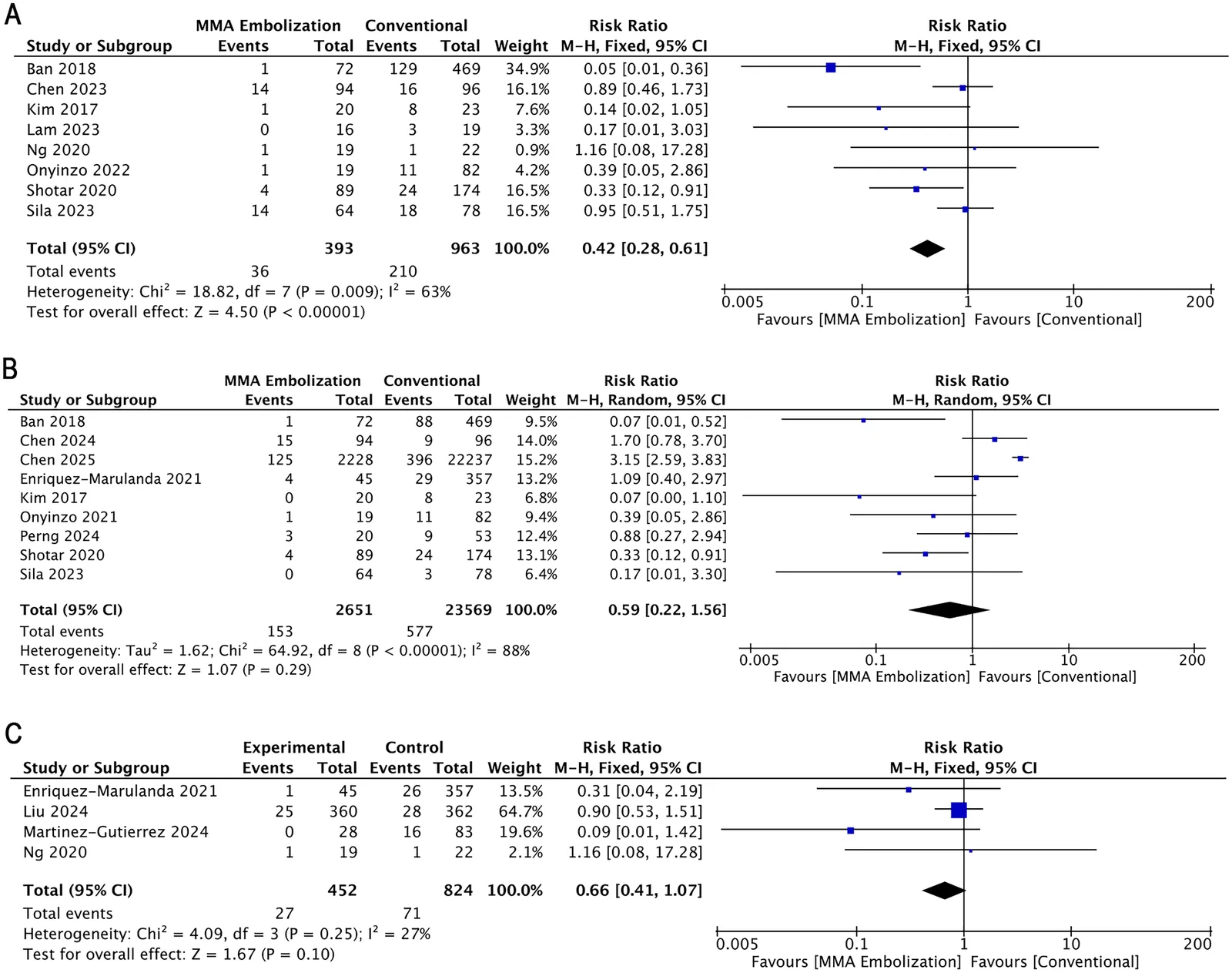
Forest plot for **(A)** treatment failure; **(B)** surgical salvage; **(C)** rehospitalization.

Eight studies reported treatment failure, with 36 events in the MMA embolization group (*n* = 393) and 210 events in the CM group (*n* = 963). The RR of treatment failure was significantly lower in the MMA embolization group than in the CM group (9.2% versus 21.8%, RR = 0.42[0.28–0.61], *P* < 0.00001, I^2^ = 63%, *P* = 0.009; Figure 4A and Supplementary Figure S4). The certainty of the evidence was low.

#### Surgical salvage

Seven studies reported surgical salvage, with 153 events in the MMA embolization group (*n* = 2,651) and 577 events in the CM group (*n* = 23,569). No difference in the RR of surgical salvage was observed between the MMA embolization group and the CM group (5.8% versus 2.4%, RR = 0.59 [0.22–1.56], *P* = 0.29, I^2^ = 88%; Figure 4B and Supplementary Figure S5). The certainty of the evidence was low.

#### Rehospitalization

Four studies reported rehospitalization, with 27 events in the MMA embolization group (*n* = 452) and 71 events in the CM group (*n* = 824). No difference in the RR of rehospitalization was observed between the MMA embolization group and the CM group (6.0% versus 8.6%, RR = 0.66 [0.41–1.07], *P* = 0.10, I^2^ = 27%; Figure 4C and Supplementary Figure S6). The certainty of the evidence was high.

#### Improvement rate

**Figure 5:**
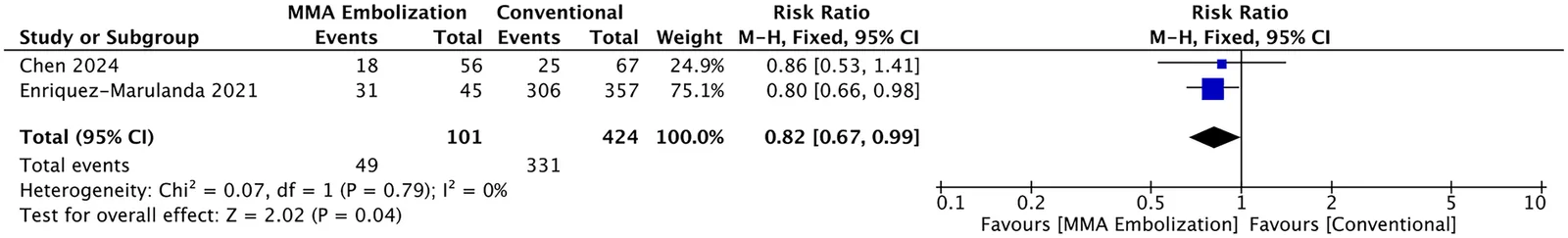
Forest plot for improvement rate.

Two studies reported improvement rates, with improvement observed in 49 cases in the MMA embolization group (*n* = 101) and 331 cases in the CM group (*n* = 424). The RR of improvement rate was significantly lower in the MMA embolization group than in the CM group (48.5% versus 78.1%, RR = 0.82 [0.67–0.99], *P* = 0.04, I^2^ = 0%; Figure 5 and Supplementary Figure S7). The certainty of the evidence was low.

## Discussion

The incidence of CSDH has been on the rise with the world's population growing older at an accelerated pace. There has been an elevation in the peak age of CSDH onset, with the highest incidence now observed in patients aged approximately 80 years (9). CSDH has become an increasingly significant neurosurgical concern, affecting 3 per 100,000 individuals (13).

Patients with CSDH may be asymptomatic or present with headache, disorientation, seizures, and speech impairment. On computed tomography (CT), CSDH commonly presents with hypodensity, isodensity, or mixed density (14).

A pathophysiological mechanism of CSDH is that inflammatory responses to subacute or chronic SDH induce the formation of vascularized membranes, which impede resorption and drive recurrence (3). Another theory holds that the outer membrane of CSDH, which originates from the dura mater, is prone to repeated microhemorrhages, thereby driving the expansion and evolution of the hematoma (15). Embolization of MMA is a therapeutic intervention designed to reduce perfusion to inflamed dural membranes (16). Ban SP states that MMA embolization aims to interrupt arterial flow from the meningeal arteries to the hematoma, effectively reducing bleeding and promoting resolution of the CSDH (15). A single-center, prospective, randomized study by Luciano Bambini Manzato et al. focusing on chronic subdural hematoma (CSDH) reported a high overall risk of CSDH recurrence, with a lower recurrence rate in the MMAE group (4.2%, *n* = 1) than in the conventional group (12.5%, *n* = 4). Their study revealed that MMAE showed a trend toward reducing the recurrence rate and 90-day mortality (17).

Another multicenter, large-scale, retrospective cohort study by Huanwen Chen et al. reported that, compared with patients receiving conventional management, patients undergoing MMAE exhibited a significantly lower risk of surgery (8.2% versus 10.9%, *P* = 0.022) and death (1.1% versus 3.0%, *P* = 0.020). Their study concluded that MMAE may benefit patients with CSDH by reducing the long-term risk of all-cause mortality (18). A prospective, multicenter, interventional, adaptive-design trial by J.M. Davies et al. showed that the recurrence rate was 4.1% in the MMAE group and 11.3% in the control (surgery alone) group (*P* = 0.008), indicating a significantly lower risk of recurrence in patients who had undergone MMAE as an adjunct to surgery for subacute or CSDH than those who had undergone surgery alone. The risk of functional deterioration was 11.9% in the MMAE group and 9.8% in the surgery alone group, indicating comparable functional deterioration in the two groups (2). A prospective study by Gahn Duangprasert et al. compared outcomes including postoperative hematoma volume, complications, and recurrence or reoperation rate between the MMAE group and the SDA (surgical drainage alone) group. Their results demonstrated a more significant reduction in hematoma volume and lower recurrence and reoperation rates in the MMAE group. Therefore, they concluded that MMAE may enhance hematoma resolution and reduce the risk of recurrence requiring reoperation (19). A multicenter, open-label, randomized trial by J. Liu et al. reported that the recurrence and aggravation rate within 90 days was 6.7% in the MMAE group and 9.9% in the control group, and the rate of serious adverse events was also lower in the MMAE group than in the control group (20). A retrospective study by Juan Carlos Martinez-Gutierrez et al. showed that the reoperation rate was significantly higher in the surgery group (16.8%) than in the adjunctive MMAE group (0.0%) (*p* = 0.006), and the recurrence rate was also significantly lower in the adjunctive MMAE group than in the surgery group (21). A study by Ying Wang et al. suggested that MMAE was associated with a decreased recurrence rate of CSDH but not with improved clinical prognosis according to the logistic analysis. They concluded that MMAE is effective in reducing the recurrence rate of CSDH (22).

Currently, other treatment methods also include Burr hole surgery (BHS) with drainage, TDC, craniotomy, and non-surgical treatments, such as corticosteroids, antifibrinolytics, and statins. Nonetheless, CSDH management is significantly challenged by the recurrence or reoperation rate varying substantially from 2% to 37% (17, 22). Effective CSDH treatment necessitates the selection of a treatment strategy tailored to the individual patient. BHD remains the preferred treatment method. Thin and small CSDHs can be managed conservatively, but the majority of cases, over 80%, often require surgical intervention.

Our review included 20 studies that examined the effectiveness of MMA embolization and CM. According to the pooled results, patients undergoing embolization of MMA exhibited a reduced rate of treatment failure, surgical salvage, and complications compared with those receiving CM. However, the resolution of symptoms was not significant among patients undergoing MMA embolization, and the improvement rate was lower than that in the CM group (48.5% versus 78.1%). Furthermore, the two groups exhibited similar mortality and mean hospital LOS.

Alejandro Enriquez-Marulanda et al. have noted that while radiological improvement following endovascular treatment may require a longer time to become manifest, the two groups exhibited comparable clinical improvement relative to radiological improvement at the final follow-up (MMA embolization versus CM: 83.3% versus 82.6%; *P* = 0.95) (23). CSDH is reported to recur in 11% to 28% of patients following burr-hole surgery. Embolization of MMA effectively disrupts the pathogenic cycle involving neomembrane formation, increased vascular permeability, inflammatory exudate accumulation, and recurrent microhemorrhages. A drawback of MMA embolization is the delayed resolution of symptoms, particularly among those affected by recurrent or refractory CSDH. Kim et al. have reported a recurrence rate varying substantially from 9.2% to 26.5%, while another study has reported an overall recurrence rate of 12.2% (10.5% among men versus 20% among women) (7, 24). J. Liu et al. have reported similar 90-day hematoma recurrence or progression rates between the MMA embolization group and the CM group, while MMA embolization is associated with a lower incidence of serious adverse events, including death (20).

In this meta-analysis, recurrence was observed in 62 patients from the MMA embolization group (*n* = 1,046) and 238 patients from the CM group (*n* = 1,547), suggesting a significantly lower RR of recurrence rate following MMA embolization versus CM (5.9% versus 15.4%, RR = 0.43 [0.32,0.58], I^2^ = 32%) (2, 7, 19–23, 25–27). Luciano Bambini Manzato et al. have reported a 3-month recurrence rate of 4.2% (*n* = 1) in the embolization group and 12.5% (*n* = 4) in the control group (absolute risk reduction: 8.3%, *P* = 0.379) (17). Moreover, lower recurrence rates and 90-day mortality have been linked to the embolization of MMA.

Regarding mortality, 65 and 4,633 deaths were recorded in the MMA embolization (*n* = 3,274) and CM (*n* = 71,522) groups, respectively, suggesting comparable mortality risks (2.0% versus 6.4%, RR = 0.98 [0.75–1.26], *P* = 0.86, I^2^ = 68%) (18, 26, 28–32). A study by J. Liu et al. has reported a lower 90-day mortality in the embolization group (0.6%) than in the CM group (2.2%) (RR = 0.27; 95% CI, 0.06–1.25) and a lower incidence of serious adverse events (including death) associated with MMA embolization (20).

### Limitations

Several limitations in this study should be noted. First, stratification of the outcomes by intervention, for instance, adjunctive or standalone embolization, is absent in the included studies. Second, subgroup analysis is infeasible because some data cannot be further obtained and analyzed.

## Conclusion

Embolization of MMA exhibits promising effectiveness for CSDH and appears potentially superior to CM.

Embolization of MMA may be particularly effective for recurrent or refractory CSDH. However, for patients with pronounced mass effect and severe brain compression, immediate relief of the hematoma-induced mass effect should be the top priority. For these cases, MMA embolization may not be the first choice; instead, BHD should be the preferred option. Among patients with comorbidities such as diabetes, hypertension, or those using anticoagulant medications, a combination of BHD and MMA embolization can be employed to prevent recurrence. The treatment of CSDH should be individualized to optimize patient outcomes.

## Data Availability

The original contributions presented in the study are included in the article/Supplementary Material, further inquiries can be directed to the corresponding author.
