# Supplementary material for: Embolizing the middle meningeal artery to treat chronic subdural hematoma: a systematic review and meta-analysis
Source: Front Surg. 2026 Aug 21;13:1941903. doi: 10.3389/fsurg.2026.1941903 (PMC13541657; doi:10.3389/fsurg.2026.1941903)
Supplement: Supplementary file 1 [file Table1.docx]

Table S1 Search strategy and result

| Pubmed | | |
| --- | --- | --- |
| # | Query | Results |
| #1 | Meningeal Arteries[MeSH Terms] | 1,254 |
| #2 | "meningeal artery"[Title/Abstract] OR "Meningeal Arteries"[Title/Abstract] OR "arteria meningealis"[Title/Abstract] OR "MMA"[Title/Abstract] | 9,219 |
| #3 | Hematoma, Subdural[MeSH Terms] | 10,530 |
| #4 | "Traumatic Subdural Hematoma"[Title/Abstract] OR "subepidural hematoma"[Title/Abstract] OR "subepidural haematoma"[Title/Abstract] OR "subdural hemorrhage"[Title/Abstract] OR "Subdural Hematoma"[Title/Abstract] OR "subdural haemorrhage"[Title/Abstract] OR "subdural haematoma"[Title/Abstract] OR "subdural bleeding"[Title/Abstract] OR "refractory subdural hematoma"[Title/Abstract] OR "pachymeningitis haemorrhagica"[Title/Abstract] OR "pachymeningiosis haemorrhagica interna"[Title/Abstract] OR "intracranial subdural hematoma"[Title/Abstract] OR "intracranial subdural haematoma"[Title/Abstract] OR "hemorrhagic pachymeningitis"[Title/Abstract] OR "haemorrhagic pachymeningitis"[Title/Abstract] OR "cSDHs"[Title/Abstract] OR "chronic subdural hematoma"[Title/Abstract] OR "chronic subdural haematomata"[Title/Abstract] OR "chronic subdural haematoma"[Title/Abstract] | 11,117 |
| #5 | Embolization, Therapeutic[MeSH Terms] | 51,881 |
| #6 | "transcatheter embolization"[Title/Abstract] OR "transcatheter embolisation"[Title/Abstract] OR "therapeutic occlusion"[Title/Abstract] OR "Therapeutic Embolizations"[Title/Abstract] OR "therapeutic embolization"[Title/Abstract] OR "intravascular embolization therapy"[Title/Abstract] OR "endovascular embolization therapy"[Title/Abstract] OR "Embolotherapy"[Title/Abstract] OR "Embolotherapies"[Title/Abstract] OR "embolization"[Title/Abstract] OR "embolisation"[Title/Abstract] OR "artificial thrombus"[Title/Abstract] OR "artificial embol*"[Title/Abstract] | 65,688 |
| #7 | ((#1 OR #2) AND (#3 OR #4)) AND (#5 OR #6) | 530 |

| Embase | | |
| --- | --- | --- |
| # | Query | Results |
| #1 | 'meningeal artery'/exp OR 'meningeal artery' | 3555 |
| #2 | 'meningeal artery':ti,ab,kw OR 'meningeal arteries':ti,ab,kw OR 'arteria meningealis':ti,ab,kw OR 'mma':ti,ab,kw | 11764 |
| #3 | 'subdural hematoma'/exp | 25737 |
| #4 | 'traumatic subdural hematoma':ti,ab,kw OR 'subepidural hematoma':ti,ab,kw OR 'subepidural haematoma':ti,ab,kw OR 'subdural hemorrhage':ti,ab,kw OR 'subdural hematoma':ti,ab,kw OR 'subdural haemorrhage':ti,ab,kw OR 'subdural haematoma':ti,ab,kw OR 'subdural bleeding':ti,ab,kw OR 'refractory subdural hematoma':ti,ab,kw OR 'pachymeningitis haemorrhagica':ti,ab,kw OR 'pachymeningiosis haemorrhagica interna':ti,ab,kw OR 'intracranial subdural hematoma':ti,ab,kw OR 'intracranial subdural haematoma':ti,ab,kw OR 'hemorrhagic pachymeningitis':ti,ab,kw OR 'haemorrhagic pachymeningitis':ti,ab,kw OR 'csdhs':ti,ab,kw OR 'chronic subdural hematoma':ti,ab,kw OR 'chronic subdural haematomata':ti,ab,kw OR 'chronic subdural haematoma':ti,ab,kw | 15332 |
| #5 | 'artificial embolization'/exp | 133570 |
| #6 | 'transcatheter embolization':ti,ab,kw OR 'transcatheter embolisation':ti,ab,kw OR 'therapeutic occlusion':ti,ab,kw OR 'therapeutic embolizations':ti,ab,kw OR 'therapeutic embolization':ti,ab,kw OR 'intravascular embolization therapy':ti,ab,kw OR 'endovascular embolization therapy':ti,ab,kw OR 'embolotherapy':ti,ab,kw OR 'embolotherapies':ti,ab,kw OR 'embolization':ti,ab,kw OR 'embolisation':ti,ab,kw OR 'artificial thrombus':ti,ab,kw OR 'artificial embol*':ti,ab,kw | 100394 |
| #7 | (#1 OR #2) AND (#3 OR #4) AND (#5 OR #6) | 743 |

| Cochrane Library | | |
| --- | --- | --- |
| # | Query | Results |
| #1 | MeSH descriptor: [Meningeal Arteries] explode all trees | 24 |
| #2 | ('meningeal artery' OR 'Meningeal Arteries' OR 'arteria meningealis' OR 'MMA'):ti,ab,kw | 489 |
| #3 | MeSH descriptor: [Hematoma, Subdural] explode all trees | 242 |
| #4 | ('Traumatic Subdural Hematoma' OR 'subepidural hematoma' OR 'subepidural haematoma' OR 'subdural hemorrhage' OR 'Subdural Hematoma' OR 'subdural haemorrhage' OR 'subdural haematoma' OR 'subdural bleeding' OR 'refractory subdural hematoma' OR 'pachymeningitis haemorrhagica' OR 'pachymeningiosis haemorrhagica interna' OR 'intracranial subdural hematoma' OR 'intracranial subdural haematoma' OR 'hemorrhagic pachymeningitis' OR 'haemorrhagic pachymeningitis' OR 'cSDHs' OR 'chronic subdural hematoma' OR 'chronic subdural haematomata' OR 'chronic subdural haematoma'):ti,ab,kw | 823 |
| #5 | MeSH descriptor: [Embolization, Therapeutic] explode all trees | 1314 |
| #6 | ('transcatheter embolization' OR 'transcatheter embolisation' OR 'therapeutic occlusion' OR 'Therapeutic Embolizations' OR 'therapeutic embolization' OR 'intravascular embolization therapy' OR 'endovascular embolization therapy' OR 'Embolotherapy' OR 'Embolotherapies' OR 'embolization' OR 'embolisation' OR 'artificial thrombus' OR 'artificial embol*'):ti,ab,kw | 6342 |
| #7 | ((#1 OR #2) AND (#3 OR #4)) AND (#5 OR #6) | 61 |

| Web of Science | |
| --- | --- |
| Query | Results |
| TS=("Traumatic Subdural Hematoma" OR "subepidural hematoma" OR "subepidural haematoma" OR "subdural hemorrhage" OR "Subdural Hematoma" OR "subdural haemorrhage" OR "subdural haematoma" OR "subdural bleeding" OR "refractory subdural hematoma" OR "pachymeningitis haemorrhagica" OR "pachymeningiosis haemorrhagica interna" OR "intracranial subdural hematoma" OR "intracranial subdural haematoma" OR "hemorrhagic pachymeningitis" OR "haemorrhagic pachymeningitis" OR "cSDHs" OR "chronic subdural hematoma" OR "chronic subdural haematomata" OR "chronic subdural haematoma") | 9964 |
| TS=("transcatheter embolization" OR "transcatheter embolisation" OR "therapeutic occlusion" OR "Therapeutic Embolizations" OR "therapeutic embolization" OR "intravascular embolization therapy" OR "endovascular embolization therapy" OR "Embolotherapy" OR "Embolotherapies" OR "embolization" OR "embolisation" OR "artificial thrombus" OR "artificial embol*" ) | 71528 |
| TS=("meningeal artery" OR "Meningeal Arteries" OR "arteria meningealis" OR "MMA") | 21185 |
| #1 AND #2 AND #3 | 499 |
