# Supplementary material for: Embolizing the middle meningeal artery to treat chronic subdural hematoma: a systematic review and meta-analysis
Source: Front Surg. 2026 Aug 21;13:1941903. doi: 10.3389/fsurg.2026.1941903 (PMC13541657; doi:10.3389/fsurg.2026.1941903)
Supplement: Supplementary file 2 [file Table2.docx]

**Table S2** Quality of Evidence was evaluated via the Grading of Recommendations Assessment, Development, and Evaluation (GRADE) approach.

**Author(s):**

**Question:** Middle meningeal artery embolization compared to conventional management for chronic subdural hematoma

**Setting:**

**Bibliography:**

| **Certainty assessment** | | | | | | | **№ of patients** | | **Effect** | | **Certainty** | **Importance** |
| --- | --- | --- | --- | --- | --- | --- | --- | --- | --- | --- | --- | --- |
| **№ of studies** | **Study design** | **Risk of bias** | **Inconsistency** | **Indirectness** | **Imprecision** | **Other considerations** | **middle meningeal artery embolization** | **conventional management** | **Relative (95% CI)** | **Absolute (95% CI)** |  |  |
| **Recurrence Rate** | | | | | | | | | | | | |
| 12 | non-randomised studies | not serious | not serious | not serious | not serious | strong association | 62/1043 (5.9%) | 238/1555 (15.3%) | **RR 0.43** (0.32 to 0.57) | **87 fewer per 1,000** (from 104 fewer to 66 fewer) | ⨁⨁⨁⨁ High |  |
| **Complication** | | | | | | | | | | | | |
| 14 | non-randomised studies | not serious | not serious | not serious | not serious | none | 122/1283 (9.5%) | 16187/50264 (32.2%) | **RR 0.91** (0.77 to 1.08) | **29 fewer per 1,000** (from 74 fewer to 26 more) | ⨁⨁◯◯ Low |  |
| **Mortality Rate** | | | | | | | | | | | | |
| 10 | non-randomised studies | not serious | not serious | not serious | not serious | none | 65/2578 (2.5%) | 4633/54177 (8.6%) | **RR 0.72** (0.55 to 0.94) | **24 fewer per 1,000** (from 38 fewer to 5 fewer) | ⨁⨁⨁⨁ High |  |
| **treatment failure rate** | | | | | | | | | | | | |
| 8 | non-randomised studies | not serious | not serious | not serious | not serious | none | 36/415 (8.7%) | 210/976 (21.5%) | **RR 0.40** (0.18 to 0.87) | **129 fewer per 1,000** (from 176 fewer to 28 fewer) | ⨁⨁◯◯ Low |  |
| **Surgical rescue** | | | | | | | | | | | | |
| 9 | non-randomised studies | not serious | not serious | not serious | not serious | none | 153/1942 (7.9%) | 577/6184 (9.3%) | **RR 0.60** (0.33 to 1.09) | **37 fewer per 1,000** (from 63 fewer to 8 more) | ⨁⨁◯◯ Low |  |
| **rehospitalization** | | | | | | | | | | | | |
| 4 | non-randomised studies | not serious | not serious | not serious | not serious | very strong association | 27/460 (5.9%) | 71/839 (8.5%) | **RR 0.66** (0.41 to 1.06) | **29 fewer per 1,000** (from 50 fewer to 5 more) | ⨁⨁⨁⨁ High |  |
| **improvement** | | | | | | | | | | | | |
| 2 | non-randomised studies | not serious | not serious | not serious | not serious | none | 44/92 (47.8%) | 38/81 (46.9%) | **RR 1.05** (0.79 to 1.41) | **23 more per 1,000** (from 99 fewer to 192 more) | ⨁⨁◯◯ Low |  |

**CI:** confidence interval; **RR:** risk ratio
