# Supplementary material for: Embolizing the middle meningeal artery to treat chronic subdural hematoma: a systematic review and meta-analysis
Source: Front Surg. 2026 Aug 21;13:1941903. doi: 10.3389/fsurg.2026.1941903 (PMC13541657; doi:10.3389/fsurg.2026.1941903)
Supplement: Supplementary file 3 [file Table3.docx]

**Table S3** Characteristics of Included Studies.

| No | First Author | Publication Year | Country | Study Design | Sample Size | Age(mean ± SD/Median (IQR)) | | | | Male, n (%) | | Trauma history (%) | | | Antiplatelet or anticoagulant use | | | | Hematoma side | | | Hematoma width (mm)(Mean±SD) | |
| --- | --- | --- | --- | --- | --- | --- | --- | --- | --- | --- | --- | --- | --- | --- | --- | --- | --- | --- | --- | --- | --- | --- | --- |
|  |  |  |  |  | Embolization | Control group | Embolization | Control group | Embolization | | Control group | | Embolization | Control group | | Embolization | Control group | Embolization | | Control group | Embolization | | Control group |
| 1 | Liu | 2024 | China | RCT | 360 | 362 | 68.5 (60–74) | 70 (61–75) | 292 (81.1) | | 304 (84.0) | | 178 (49.4) | 201 (55.5) | | 28 (7.8) | 25 (6.9) | NR | | NR | NR | | NR |
| 2 | Ban | 2018 | Korea | Prospective- retrospective cohort | 72 | 469 | 69.3±10.47 | 67.4±13.16 | 48 (66.7) | | 347 (74.0) | | 45 (62.5) | 272 (58.0) | | 29 (40.3) | 121 (25.8) | Right:25 (34.7); Left: 28 (38.9); Bilatera:19 (26.4) | | Right:149 (31.8); Left:213 (45.4); Bilatera:107 (22.8) | 19.6 (5.00) | | 20.3 (6.47) |
| 3 | Shotar | 2020 | France | monocentric retrospective study | 89 | 174 | 74±13 | 77±12 | 68 (76) | | 123 (71) | | NR | NR | | Antiplatelets:49% (44); Anticoagulants:30% (27) | Antiplatelets:55% (96); Anticoagulant:31% (54) | NR | | NR | NR | | NR |
| 4 | McCann | 2023 | USA | retrospective cohort | 245 | 48316 | 67.1±17.1 | 68.4±15.9 | 169(69) | | 32949(68.2) | | 142 (58.0%) | 29566 (61.2%) | | 33 (13.5%) | 7534 (15.6% | NR | | NR | NR | | NR |
| 5 | Sila | 2023 | Germany | retrospective cohort | 64 | 78 | 75±12 | 78±10 | 48(75) | | 60(76.9) | | NR | NR | | Antiplatelets:26.5% (17); Anticoagulants:29.7% (19) | Antiplatelets:25.6 %(20); Anticoagulants:26.9%(21) | Right: 19 (29.7%); Left: 28 (43.8%) ; Bilatera: 17 (26.6%) | | Right: 30 (38.5%); Left: 30(38.5%); Bilatera:18 (23.1%) | 18(6) | | 21(5) |
| 6 | Kim | 2017 | Korea | retrospective cohort | 20 | 23 | 73.65±7.14 | 65.65±6.91 | 14(70.0%) | | 16(69.6%) | | 8(40.0%) | 17(73.9%) | | Antiplatelets:6 (30.0); Anticoagulants:3 (15.0) | Antiplatelets:1 (4.3); Anticoagulants: 1 (4.3) | NR | | NR | NR | | NR |
| 7 | Housley | 2023 | USA | retrospective cohort | 44 | 48 | 73.3±11.9 | 71.1±13.2 | 25(56.8) | | 21(43.8) | | NR | NR | | 21(47.7) | 28(58.3) | NR | | NR | 16(5.8) | | 16.4(9.6) |
| 8 | Enriquez-Marulanda | 2021 | USA | retrospective cohort | 45 | 357 | 76(69-87) | 74(65-82) | 28 (62.2%) | | 247 (69.2%) | | NR | NR | | Antiplatelets:24(53.3%); Anticoagulant10(22.2%) | Antiplatelets:115 (32.2%); Anticoagulants: 70 (19.6%) | Right:19 (42.2%); Left: 26 (57.8%) | | Right: 169 (47.3%); Left: 188 (52.7%) | 18(13-23.4mm) | | 18(12.3-24.2) |
| 9 | Chen | 2025 | USA | retrospective cohort | 2228 | 22237 | 71(62-78) | 71(60-80) | 1604(72.0) | | 14101(63.4) | | NR | NR | | Antiplatelets:18.2% (405); Anticoagulants:24.9% (555) | Antiplatelets:19.3% (4292); Anticoagulants:23.9% (5320) | NR | | NR | NR | | NR |
| 10 | Duangprasert | 2024 | Thailand | prospective, non-randomized, single-center study | 20 | 23 | 68.2 ± 8.7 | 70.2 ± 11.1 | 17 (85.0) | | 18 (78.3) | | NR | NR | | 8(40%) | 7(30.4%) | Right: 7 (50%); Left:7 (26.9%); Bilateral:6 (23.1%) | | Right: 11(53.9%); Left:9 (34.6%); Bilateral, 3 (11.5%) | 18.1±6.3 | | 19.8 ±5.6 |
| 11 | Lam | 2023 | Australia | RCT | 16 | 19 | 64.2 | 72.4 | 12(75.0) | | 11(57.9) | | 13(81.2) | 16(84.2) | | Antiplatelets:2 (12.5); Anticoagulants:1 (6.25) | Antiplatelets:2 (10.5); Anticoagulants:2 (10.5) | NR | | NR | 21.1 | | 20.9 |
| 12 | Perng | 2024 | Taiwan | retrospective cohort | 20 | 53 | 70.32 (63.3, 78.0) | 75.93 (69.5, 80.4) | 18.7 (92.1) | | 38.8 (73.8) | | NR | NR | | Antiplatelet :6.3(31.3%); anticoagulant:1.9(9.3%) | Antiplatelet :23.5(44.8%); anticoagulant:1.7(3.3%) | NR | | NR | 17.69 (13.41, 22.38) | | 16.15 (14.00, 21.60) |
| 13 | Shotar | 2025 | France | RCT | 171 | 171 | 77 (68-83) | 77 (69-84) | 139 (81.3) | | 135 (78.9) | | 69/144(47.9) | 70/141(49.6) | | Antiplatelet /anticoagulant:119(69.6%) | Antiplatelet /anticoagulant:118(69.0%) | NR | | NR | unilateral:22(18-26);bilateral:22(17-26) | | unilateral:23(20-26);bilateral:23(15.2-26) |
| 14 | Wang | 2024 | China | monocentric retrospective study | 23 | 205 | 71.7 ± 8.9 | 67.6±11.2 | 15 (65.2) | | 162 (79.0%) | | 9 (39.1) | 82(40.0%) | | Use of anticoagulant or antiplatelet11(47.8%) | 46(22。4%) | NR | | NR | 22.4 ± 4.6 | | 19.8 ± 12.9 |
| 15 | Davies | 2024 | USA | RCT | 197 | 203 | 73.0±11.0 | 71.0±11.3 | 143 (72.6) | | 149 (73.4) | | NR | NR | | 75 (38.1) | 79 (38.9) | Right:100 (50.8); Left: 97 (49.2) | | Right: 92 (45.3); Left: 111 (54.7) | 21.6±6.3 | | 21.4±6.2 |
| 16 | Manzato | 2025 | Brazil | RCT | 33 | 43 | 73.3±10.9 | 74.1±10.8 | 28 (84.8%) | | 32 (74.4%) | | NR | NR | | Antiplatelet :2(6.1%);anticoagulant:4(12.2%) | Antiplatelet :4(9.3%);anticoagulant:4(9.4%) | Right:13 (39.4%); Left: 20 (60.6%) | | Right: 17 (39.5%); Left: 26 (60.5%) | 20.0 (15.0 −25.0) | | 20.5 (13.8–25.3) |
| 17 | Onyinzo | 2021 | Germany | retrospective cohort | 19 | 82 | 78.2±7.5 | 77.4±10.3 | 14 (73.7%) | | 53 (64.6%) | | NR | NR | | 16 (84.2%) | 30 (36.6%) | NR | | NR | 19.2±4.4 | | 23.5±4.7 |
| 18 | Martinez-Gutierrez | 2024 | USA | a single center, retrospective cohort study | 28 | 83 | 74 (68− 81) | 73 | 22 (78.6) | | 67 (80.7) | | NR | NR | | Antiplatelets:4 (14.3); Anticoagulants:3 (10.7) | Antiplatelets:30 (36.1); Anticoagulants:10 (12.0) | NR | | NR | 21.4±5.1 | | 20.2±6.1 |
| 19 | Ng | 2020 | France | single-center, prospective, randomized, pilot study | 19 | 22 | 77.4±10.9 | 74.7±13.9 | 10 (52.6) | | 13 (59.1) | | 12 (63) | 15 (68) | | 7 (37) | 9 (41) | Right:8 (42); Left: 8 (42); Bilatera:3 (16) | | Right: 10 (45); Left:9 (41); Bilatera:3 (14) | 14.7±5.4 | | 13.6±4.7 |
| 20 | Chen | 2024 | USA | retrospective study | 94 | 96 | 73 (65–80) | 71 (62–82) | 78 (83.0) | | 80 (83.3) | | NR | NR | | Antiplatelets:35 (37.2); Anticoagulants:22 (23.4) | Antiplatelets:35 (36.4); Anticoagulants:21 (21.9) | left:57/115(49.6) | | left:54/115(47.0) | 16 (12–20) | | 15 (12–21) |
