# Supplementary material for: Embolizing the middle meningeal artery to treat chronic subdural hematoma: a systematic review and meta-analysis
Source: Front Surg. 2026 Aug 21;13:1941903. doi: 10.3389/fsurg.2026.1941903 (PMC13541657; doi:10.3389/fsurg.2026.1941903)
Supplement: Supplementary file 4 [file Table4.docx]

**Table S4** Newcastle Ottawa Scale assessment of non-randomized studies.

| Study | Selection | | | | Comparability | Outcome | | | Quality scores |
| --- | --- | --- | --- | --- | --- | --- | --- | --- | --- |
|  | Representativeness of the exposed cohort | Selection of the nonexposed cohort | Ascertainment of exposure | Demonstration that outcome of interest wae not present at start og study | Comparability of cohorts on the basis of the design or analysis | Assessment of outcome | Was follow up long enough for outcomes to occur | Adequacy of follow up of cohorts |  |
| Ban 2018 | * | * |  | * | * | * |  | * | 6 |
| Chen 2024 | * | * | * | * |  | * | * | * | 7 |
| Chen 2025 | * | * | * | * | ** | * | * | * | 9 |
| Duangprasert 2024 | * | * | * | * | * |  | * | * | 7 |
| Enriquez-Marulanda 2021 | * | * | * | * | * | * | * | * | 8 |
| Housley 2022 | * | * | * | * | * | * | * | * | 8 |
| Kim 2017 | * | * | * | * | * |  |  | * | 6 |
| Martinez-Gutierrez 2024 | * | * | * | * | * | * | * | * | 8 |
| McCann 2023 | * | * | * | * | * |  | * | * | 7 |
| Onyinzo 2021 | * | * | * | * |  |  |  | * | 5 |
| Perng 2024 | * | * | * | * | * | * | * |  | 7 |
| Shotar 2020 | * | * | * | * | * |  | * | * | 7 |
| Sila 2023 | * | * | * | * | * | * |  | * | 7 |
| Wang 2024 | * | * | * | * | * |  | * | * | 7 |
