# Supplementary material for: Embolizing the middle meningeal artery to treat chronic subdural hematoma: a systematic review and meta-analysis
Source: Front Surg. 2026 Aug 21;13:1941903. doi: 10.3389/fsurg.2026.1941903 (PMC13541657; doi:10.3389/fsurg.2026.1941903)
Supplement: Supplementary file 5 [file Table5.docx]

**Table S5** Details of PICO and follow-up duration in each included study.

| No | First Author | Publication Year | P | I | C | O | Follow-up (mo) |
| --- | --- | --- | --- | --- | --- | --- | --- |
|  |  |  |  |  |  |  |  |
| 1 | Liu | 2024 | Adult patients (≥18y) with symptomatic nonacute SDH (subacute/chronic) demonstrating mass effect. | MMAE was performed under general/monitored local anesthesia via femoral/radial access using Onyx to embolize the MMA trunk or frontal/parietal branches. Successful embolization required target vessel occlusion, with most procedures preceding burr-hole drainage. | Patients who were assigned to the usual-care group were treated according to the current standard of care — that is, burr-hole drainage as needed, best medical management, or both. | Symptomatic recurrence or progression of subdural hematoma, late symptomatic recurrence or progression of the subdural hematoma at 1 year, EuroQol Group 5-Dimension 5-Level SelfReport Questionnaire (EQ-5D-5L), serious adverse events, complications. | 3 months, 1 year |
| 2 | Ban | 2018 | CSDH patients (≥20y) were enrolled, excluding those with: focal lesions, thickness ≤10mm, no mass effect, underlying pathology (e.g., vascular/tumor), or life expectancy <6 months. | MMA embolization was performed under local anesthesia using 150-250 μm PVA particles, with microcatheter-based branch embolization (convexity dura supply) without heparin. Asymptomatic cases received embolization alone; symptomatic cases had additional hematoma evacuation. | Conventional treatment comprised: 1) surgical evacuation (burr-hole/craniotomy) for symptomatic cases, or 2) surveillance CT every 2-4 weeks for asymptomatic patients, with delayed surgery if progression occurred. | Treatment failure,surgical rescue,complications. | 1, 3, and 6 months |
| 3 | Shotar | 2020 | Patients were eligible if operated by single burr-hole craniostomy for a CSDH recurrence or a CSDH in the case of an independent risk factor defined as follows: (1) antiplatelet therapy11 or (2) full anticoagulation therapy or (3) coagulation disorder or (4) hepatopathy or (5) chronic alcoholism. | Embolization was performed with calibrated triacryl gelatin microspheres (300 to 500 µm in diameter) in 81 instances, with associated proximal MMA coiling in 27 of the 81 cases, using an n-­butyl cyanoacrylate liquid embolic agent in five cases, and by proximal MMA coiling alone in the remaining five cases. | Single burr-hole craniostomy ± closed-system drainage. | Recurrence rate, complications. | 3 months |
| 4 | McCann | 2023 | Adult patients (≥18 years) diagnosed with chronic subdural hematoma (cSDH), with exclusion of those with aneurysms, neoplasms, precerebral arteriopathies, or dissections. | Middle meningeal artery embolization (MMAe), a minimally invasive endovascular procedure identified via specific ICD codes (ICD-9 39.72, 39.75, 39.76; ICD-10 03LG, 03LM, 03LN), performed to treat cSDH. | Conservative management (no intervention) and traditional surgical treatment for cSDH (identified via previously published ICD codes for procedures such as craniotomy or burr hole evacuation) | Cost of admission, hospital length of stay (LOS), nonroutine discharge disposition (destination other than home), and complications. | / |
| 5 | Sila | 2023 | Adult patients with a unilateral or bilateral chronic subdural hematoma seen in an admission CT scan. | MMA embolization was performed under general anesthesia via femoral access, with microcatheter-guided PVA particle (150-250 μm) embolization of target branches, followed by 72-hour intermediate care monitoring. | Surgical evacuation via burr hole or small craniotomy under general anesthesia, with Ringer's solution irrigation and 3-day subdural drainage. | Treatment failure, secondary treatment of the treatment failure cases, median number of CT scans during follow-up, mean follow-up in days, reatment-related complications, Variables predicting treatment failure. | 1 and 3 months |
| 6 | Kim | 2017 | Patients with refractory (intractable) chronic subdural hematoma (CSDH) who had recurrence after initial treatment. | Perioperative embolization of the middle meningeal artery (MMA) using polyvinyl alcohol particles (150-250 μm) to occlude the frontal and parietal branches of the MMA. | Conventional treatment: reoperation (reopening the burr hole for hematoma drainage, and in some cases, additional craniostomy or craniotomy for recurrence) | Recurrence rate, functional outcome, complications, hospital stay. | 1, 3, and 6 months |
| 7 | Housley | 2022 | Patients with chronic subdural hematoma (cSDH) eligible for either standalone surgical evacuation or MMA embolization. | The intervention group underwent standalone middle meningeal artery embolization (MMAE) using Onyx as the embolic agent in all cases. This endovascular procedure aimed to devascularize the dural membranes to inhibit inflammation-driven hematoma recurrence. | The control group received surgical evacuation alone (SEA) without adjunct embolization. Surgical methods included burr hole evacuation (50%) or craniotomy (50%) for hematoma drainage. | Volumetric resolution and recurrence. | 3–12 weeks |
| 8 | Enriquez-Marulanda | 2021 | Adult patients with chronic subdural hematomas (cSDH) treated at a tertiary academic institution, excluding those with supratentorial non-convexity involvement, underlying causes (e.g., intracranial hypotension, tumors), predominant acute/subacute hematomas, unavailable pre-procedural imaging, or prior surgical treatment for the same hematoma. | Middle meningeal artery (MMA) embolization, performed either as primary treatment (77.7%) or adjunctive to planned open surgery (22.3%). Embolization techniques included polyvinyl particles (150–250 μm) with coil occlusion (95.6%) or coils alone (4.4%). | Conventional open surgical treatment (burr-hole craniostomy, craniotomy, or craniectomy) without embolization. | Complication rates, clinical improvement rates, cSDH recurrence rates, overall re-intervention rates, modified Rankin Scale (mRS) scores at last follow-up, mortality, and radiological improvement rates at first and last follow-up imaging. | 3 months |
| 9 | Chen | 2025 | Patients with nonsurgical nontraumatic subdural hematoma (SDH) identified from the 2019–2021 Nationwide Readmissions Database (NRD), excluding those who underwent upfront surgical evacuation, had do-not-resuscitate (DNR) status, transitioned to comfort care, died during initial hospitalization, or had duplicate records. | Standalone middle meningeal artery embolization (MMAE) performed without concurrent surgical evacuation during the initial hospitalization. | Conservative management (CM) involving noninvasive, expectant monitoring without surgical or endovascular intervention. | Surgical rescue, mortality. | 1 and 3 months |
| 10 | Duangprasert | 2024 | Symptomatic adults (≥18y) with CSDH requiring surgical drainage were included, excluding: prior CSDH surgery, >50% acute component, secondary causes (e.g., vascular lesions), life expectancy <6 months, asymptomatic cases, or dangerous anastomoses. Bilateral cases were evaluated per side. | Adjunctive middle meningeal artery embolization (MMAE) performed within 7 days after surgical drainage (burr hole evacuation with subdural drain placement). Embolization used 100–300 µm Embosphere® microspheres under fluoroscopic guidance, targeting distal MMA branches or the main trunk. | Surgical drainage alone (SDA) without embolization, involving burr hole evacuation with subdural drain placement (removed within 72 hours). | Recurrence rate, complications, hospital stay, and functional status. | 1, 3, and 6 months |
| 11 | Lam | 2023 | Patients with septated cSDH undergoing surgical evacuation, either as standalone procedure or combined with MMAE. | Adjuvant middle meningeal artery embolization (MMAE) was performed after surgical evacuation of septated cSDH, with the procedure involving the use of embolization materials such as coils, liquid embolics, particles, or combinations thereof. | The comparison is with surgical evacuation alone, which includes procedures such as burr hole evacuation or craniotomy without subsequent MMAE, with postoperative subdural drain placement as part of the institutional practice. | Surgical reoperation rate, and secondary outcomes such as recurrence, changes in hematoma thickness, changes in midline shift on follow-up imaging, and procedure-related complications. | 6 months |
| 12 | Perng | 2024 | CSDH patients treated with surgical evacuation or MMAE with simple coiling were included. Inclusion required: (1) CT-confirmed diagnosis, (2) age ≥20 years, and (3) follow-up CT available. Exclusions: intracranial tumors, leptomeningeal disease, shunt-related CSDH, or missing follow-up imaging. | MMAE via femoral approach under local anesthesia, with main MMA trunk coiling confirmed by post-procedural angiography (absence of contrast filling). | CSDH evacuation via burr hole craniostomy under general anesthesia, with saline irrigation and optional subdural drain placement (burr hole number at surgeon's discretion). | Urgical rescue rate, and secondary outcomes such as hematoma thickness reduction (>50% and >70%), improvement in midline shift, and Modified Rankin Scale (mRS) scores. | 1 and 6 months |
| 13 | Shotar | 2025 | Adult patients (≥18 years) undergoing burr hole surgery for either: (1) recurrent CSDH, or (2) first-episode CSDH with high recurrence risk (antiplatelet/anticoagulant use, chronic alcoholism, cirrhosis, thrombocytopenia, or no subdural drain placement). | MMA embolization with 300-500 μm TAGM microspheres was performed within 7 days postoperatively, involving superselective catheterization beyond the foramen spinosum with free-flow particle injection, alongside standard care. | Standard care alone (without MMA embolization), with neurosurgeon-directed management including imaging, admission, and reoperation decisions. | Rate of CSDH recurrence, need for repeat surgery,repeat surgery rate, mortality, hospital stay, and embolization-related complications. | 1 and 6 months |
| 14 | Wang | 2024 | Consecutive CSDH patients undergoing BHC were included, excluding those with: (1) other intracranial pathologies, (2) severe coagulopathy/hepatorenal dysfunction, (3) incomplete imaging, or (4) lost to follow-up. | Middle meningeal artery embolization (MMAE) was performed as an adjunct postoperative treatment after BHC, involving navigation of a microcatheter into the middle meningeal artery (MMA) via femoral or radial access, selective angiography to check for collateral vessels, and embolization of target MMA branches with Onyx under local anesthesia. | Burr hole craniostomy alone (standard CSDH surgical treatment without adjunctive MMA embolization). | Recurrence rate, complication rates, adverse prognosis. | 1 and 3 months |
| 15 | Davies | 2024 | Patients aged 18-90 years requiring surgical evacuation for symptomatic subacute/chronic SDH were included, with indications including: neurologic deficits beyond headache/imbalance/confusion, focal motor deficits, hematoma thickness >15 mm, or midline shift ≥5 mm. Exclusions were: life expectancy <1 year, pre-hematoma mRS 4-5, or Markwalter score ≥3. | Adjunctive MMA embolization using Onyx was performed within 48h post-randomization (pre- or post-surgery) via biplane DSA, with microcatheter navigation to the ipsilateral MMA and Onyx injection for branch occlusion. | Surgery alone (burr hole craniostomy or craniotomy per surgeon preference) without MMA embolization. | Recurrence or progression of subdural hematoma, hospital readmissions, adverse events. | 3 and 6 months |
| 16 | Manzato | 2025 | Adult patients (>18 years) with untreated chronic subdural hematoma requiring surgical drainage. | MMAE using 14% Histoacryl-lipiodol mixture was performed within one week postoperatively, involving superselective MMA microcatheterization and main trunk occlusion under general anesthesia. | Burr hole evacuation (1 or 2 holes) with surgeon-discretionary subdural drainage, without adjunctive MMA embolization. | recurrence rate, length of hospital stay, complications, and mortality. | 1 and 3 months |
| 17 | Onyinzo | 2021 | Patients with chronic subdural hematoma (CSDH). | MMA embolization was performed through femoral access, with microcatheterization of frontal/parietal MMA branches using 45-150 µm PVA particles and/or platinum coils, either as standalone treatment or combined with burr hole irrigation. | Burr hole irrigation alone (subdural irrigation under general/local anesthesia without MMA embolization). | Treatment-related complications, clinical outcome at discharge, and the rate of revision surgery. | 3 months |
| 18 | Martinez-Gutierrez | 2024 | Patients with septated chronic subdural hematomas (cSDH) undergoing surgical evacuation were analyzed, including both surgery-alone cases and combined surgery with middle meningeal artery embolization (MMAE). | Adjuvant MMAE was performed postoperatively for septated cSDH, utilizing coils, liquid embolics, particles, or their combinations. | Surgical evacuation alone (burr hole or craniotomy without MMAE), with routine postoperative subdural drain placement per institutional protocol. | Surgical reoperation rate, recurrence, procedure-related complications. | 1 -2 months |
| 19 | Ng | 2020 | Symptomatic adult patients (≥18 years) with chronic subdural hematoma (CSDH) requiring surgical treatment were included. | Adjuvant middle meningeal artery (MMA) embolization was performed during the same hospital admission following surgical evacuation. The procedure involved selective microcatheterization of MMA branches and embolization using 150-250 μm polyvinyl alcohol particles under conscious sedation. | Standalone surgical treatment (twist-drill craniostomy or wide craniotomy with subdural drain removed within 48 hours) was performed without MMA embolization. | CSDH recurrence, CSDH recurrence, hematoma volume resorption (HVR) at 3 months. | 3 months |
| 20 | Chen | 2024 | Adult patients with chronic subdural hematomas (cSDHs) who underwent successful middle meningeal artery embolization (MMAE) at 14 high-volume centers in the US. | MMAE with concurrent surgical evacuation, defined as patients undergoing both MMAE and surgical intervention (burr hole craniostomy, craniotomy, or subdural evacuation port system placement) either during the same hospitalization or within 14 days following MMAE. | Standalone middle meningeal artery embolization (MMAE), defined as a minimally invasive endovascular procedure involving embolization of the middle meningeal artery using particles, coils, liquid embolics (e.g., Onyx or nBCA), or their combinations, performed without concurrent surgical evacuation. | CSDH recurrence, radiographic failure, death, 90-day functional outcome, procedural complications. | 3 months |
